# CT, MRI, and PET/CT imaging features of thoracic spine epithelioid hemangioma: a retrospective observational study

**DOI:** 10.3389/fonc.2024.1296401

**Published:** 2024-06-19

**Authors:** Xianwen Hu, Xiaotian Li, Zujiang Xiong, Dandan Li, Jiong Cai, Pan Wang

**Affiliations:** ^1^ Department of Nuclear Medicine, Affiliated Hospital of Zunyi Medical University, Zunyi, China; ^2^ Department of Radiology, Qianxinan People’s Hospital, Bijie, China; ^3^ Department of Radiology, Chongqing Fifth People’s Hospital, Chongqing, China; ^4^ Department of Obstetrics, Zunyi Hospital of Traditional Chinese Medicine, Zunyi, China

**Keywords:** epithelioid hemangioma, thoracic spine, computed tomography, magnetic resonance imaging, positron emission tomography

## Abstract

**Introduction:**

Epithelioid hemangioma (EH) is an intermediate locally aggressive tumor that consists of epithelioid cells and endothelial cell differentiation, which can occur at any age, but is most common between the ages of 30 and 40 years. EH in the thoracic spine is rare, and accurate diagnosis is critical to treatment planning. Our aim was to explore the imaging and clinical data of thoracic spine EH to improve the understanding of this rare disease.

**Methods:**

From January 1, 2018 to June 30, 2023, a database of thoracic spine masses was retrospectively reviewed. Five patients with histologically proven thoracic spine EH and complete imaging available were identified and analyzed. Computed tomography (CT) and magnetic resonance imaging (MRI) findings were evaluated separately by two radiologists with more than 10 years of experience. Positron emission tomography (PET)/CT was conducted by two nuclear medicine diagnostic technologists with at least 5 years of experience.

**Results:**

The patients included three male and two female patients aged 23 to 56 years (mean age was 38.4 ± 14.3 years). All patients underwent CT, MRI, and ^18^F-FDG PET/CT examination before treatment. Four patients were limited to one vertebral involvement, only one patient had multiple vertebral involvement, and all tumors involved the accessories, including one involving the posterior ribs. The maximum diameter of the tumor ranged from 2.7 to 4.3.

**Conclusions:**

CT, MRI, and ^18^F-FDG PET/CT findings of thoracic spine EH have certain characteristics, and understanding these imaging findings will help to obtain accurate diagnosis before surgery.

## Introduction

Epithelioid hemangioma (EH) of the bone has been controversial since its first report in 1969, which was previously classified as a moderately locally invasive and rarely metastatic tumor. Until 2013, the World Health Organization (WHO) defined EH as a unique solid tumor that is sometimes difficult to distinguish from epithelioid hemangioendothelioma (1). In the latest version of the WHO classification of bone and soft tissue tumors, it is uniformly classified as an intermediate locally aggressive tumor (2). EH is more common in women, with a peak onset age of 20–40 years old (3). It often occurs in the head and neck, especially around the ears, presenting as a single subcutaneous nodule or red papule-like lesion with an average diameter of less than 1 cm, with a few occurring in the lymph nodes, testes, bones, and so on (4). EH that occurs in the bone tissue is most common in the long tubular bones of the limbs, followed by the short tubular and flat bones in the distal lower limbs, and is relatively rare in the spine (5, 6). Most patients have no obvious clinical symptoms. Patients with EH that occurs in the spine may experience neurological symptoms when the tumor compresses the spinal cord (4). The treatment of epithelioid hemangioma mainly involves complete local resection, with some areas prone to recurrence. Therefore, obtaining accurate diagnosis is still crucial. In the present study, computed tomography (CT), magnetic resonance imaging (MRI), and fluoro-18 fluorodeoxyglucose (^18^F-FDG) positron emission tomography (PET)/CT imaging features of thoracic EH treated in our hospital were retrospectively analyzed, and the aim was to increase the further understanding of this rare disease and improve the correct diagnosis rate.

## Materials and methods

### Patients

The current retrospective study was approved by the institutional review board of The Affiliated Hospital of Zunyi Medical University, and written informed consent was waived.

A retrospective analysis of the database of patients with histologically confirmed thoracic vertebrae EH admitted to the affiliated hospital of Zunyi Medical University from January 1, 2018 to June 30, 2023 was performed, and the imaging features and clinical data were extracted. The inclusion criteria included patients with pathological diagnosis of thoracic spine EH and CT, MRI, and ^18^F-FDG PET/CT examinations performed before treatment. The exclusion criteria included the following: (i) the image quality is poor and cannot be analyzed correctly; and (ii) the lesion area had been subject to any treatment such as surgical resection and radiotherapy before CT, MRI, and ^18^F-FDG PET/CT examinations.

### Image acquisition

**CT.** All patients underwent CT scans from the seventh cervical spine to the first lumbar spine using a 16-row scanner from Siemens SOMATOM Sensation in Germany (Scanning parameters: tube voltage, 120 kV; tube current, 220 mAs, layer thickness, 8–10 mm). Contrast-enhanced scanning was performed using iodohexanol (300 mg I/ml) at 1.5 ml/kg, and a high-pressure syringe was used for single-phase injection at a speed of 3.0 ml/s. The arterial phase images were collected 30–36 s after injection of contrast agent, and the venous phase images were collected 60–70 s after injection of contrast agent.

**MRI.** Siemens Sensation 3.0T MR Scanner was used to perform transverse T1-weighted imaging [T1WI (TR, 100 ms; TE, 2.46 ms)], T2-weighted imaging [T2WI (TR, 1400 ms; TE, 81 ms)], and sagittal T2WI (TR, 1500 ms; TE, 80 ms). The scanning layer thickness was 6 mm, and layer spacing was 1 mm. Contrast enhanced scanning was performed by intravenous injection of gopentate meglumine 0.1 mmol/kg.

**^18^F-FDG PET/CT.** The production of ^18^F-FDG and PET/CT (Biograph mCT, Siemens, Germany) scanning parameters were conducted based on our previously published literature (7). According to patient weight, 0.1 to 0.15 mCi/kg of ^18^F-FDG was injected intravenously. Then, the patient was asked to rest quietly for 45 to 65 min, and PET/CT imaging was performed after urination.

### Image analysis

CT/MRI images were analyzed by two radiologists with more than 10 years of pertinent clinical experience. The evaluation subjects include tumor location, maximum diameter of the tumor, bone changes (including expansile osteolysis, sclerotic rim, and vertebral compression), density (compared with muscle)/signal (compared with the normal bone signal), and degree of enhancement. Visual and semiquantitative analysis of PET/CT were conducted by two nuclear medicine diagnostic technologists with at least 5 years of experience. Visual analysis of FDG uptake was categorized as higher or similar to surrounding normal bone tissue. For semi-quantitative analysis, the region of interest (ROI) was placed over the entire lesion area, and the maximum standardized uptake value (SUVmax), mean standardized uptake value (SUVmean), and metabolic tumor volume (MTV) of the lesion were calculated as evaluation subjects.

## Results

### Patient characteristics

The patient population included three male and two female patients aged 23 to 56 years (mean age was 38.4 ± 14.3 years). All patients sought medical help due to chest and back pain, with or without limb numbness. Except for one patient’s ferritin that slightly decreased, the serum tumor markers of all patients, including carbohydrate antigen-199 (Ca199), Ca125, and carcino-embryonic antigen (CEA), were within the normal range. Immunohistochemistry (IHC) results showed that CD34 (5/5) was the most frequently positive in these cases, followed by erythroblast-specific transformation-related genes (ERG) (4/5), CD31 (4/5), factor VIII (4/5), and vimentin (3/5), while all tumor cells negatively expressed transcription factor E3 (TFE3). After the diagnosis was confirmed, all patients underwent surgery to remove the mass. Thoracic spine CT or MRI was routinely examined every 6 months to 1 year after surgery, and no signs of recurrence were found during the follow-up period. The clinical and immunohistochemical features are summarized in **Table 1**.

**Table 1:** The clinical and immunohistochemical features of thoracic spine epithelioid hemangioma patients.

Case	Gender	Age	Main symptoms	Serum tumor marker	IHC	Management	Follow-up (months)
1	F	51	Chest and back pain for 2 years	Ferroprotein (-); Ca199 (-); Ca125 (-); CEA(-)	Vimentin (+); ERG (+); CD31 (+);CD34 (+); CK (-); VIII(weakly +); TFE3 (-); Ki67 (20%+)	Surgery+ radiation therapy	30/AWD
2	M	31	Chest pain with numbness for 2 months	Ferroprotein (-); Ca199 (-); Ca125 (-); CEA(-)	ERG (+); CD31 (+); CD34 (+); VIII(+); CK (-); TFE3 (-); Ki67 (10%+)	Surgery	16/AWD
3	M	56	Chest and back pain for 0.5 months	Ferroprotein (-); Ca199 (-); Ca125 (-); CEA (-)	Vimentin (+); CD31 (+); CD34 (+); FLI-1 (+); EMA (-); TFE3 (-); Ki67 (12%+)	Surgery	13/AWD
4	F	31	Shoulder and back pain with numbness in both upper limbs for 2 months	Ferroprotein (-); Ca199 (-); Ca125 (-); CEA(-)	ERG (+); CD31 (+); Vimentin (+); CD34 (+); TFE3 (-); VIII(+); CD68 (-); Ki67 (10%+)	Surgery	24/AWD
5	M	23	Chest and back pain for 1.0 months	Ferroprotein 21.0(reference: 23.9-336.2); Ca199 (-); Ca125 (-); CEA(-)	CD34 (+); TFE3 (-); ERG (+); VIII(+); FLI-1 (+); CK (-); Ki67 (5%+)	Surgery	40/AWD

M, male; F, female; AWD, alive without disease; MD, Maximum diameter; ERG, erythroblast specific transformation related genes. (−), negative; (+), positive.

### CT, MRI, and ^18^F-FDG PET/CT findings

All five patients underwent CT, MRI, and ^18^F-FDG PET/CT examination before treatment. Four patients were limited to one vertebral involvement, only one patient had multiple vertebral involvement, and all tumors involved the accessories, including one involving the posterior ribs.

### CT findings

**Figure 1:**
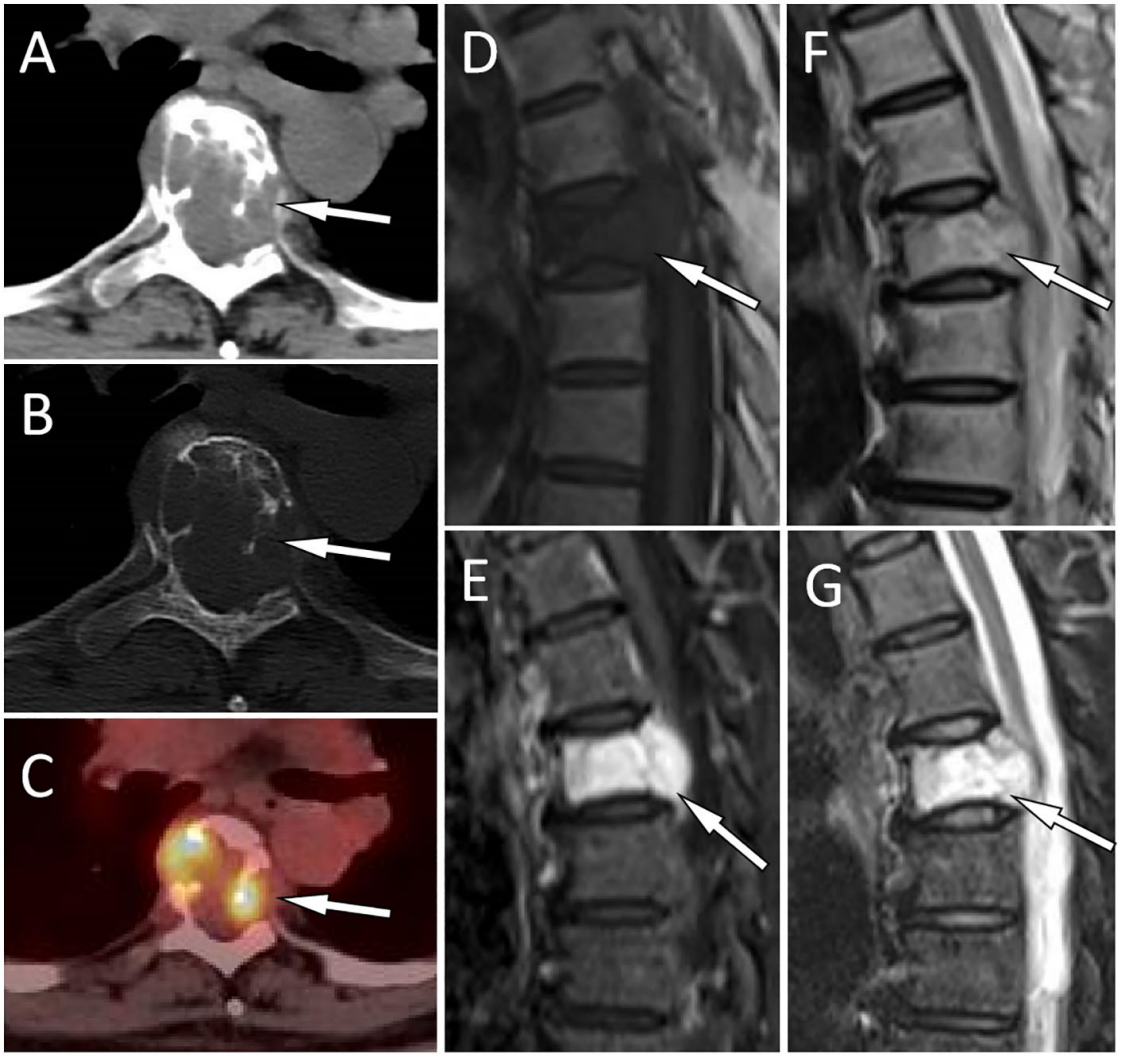
A 51-year-old woman with EH (case 1): The axial CT soft tissue window shows a soft tissue mass extending toward the periphery of the vertebral body and the left accessory as well as the spinal canal with T5 as the center [**(A)**, arrow]. The CT bone window shows expansive osteolytic bone destruction in the T5 vertebral body and the left accessory, with residual bone trabeculae visible inside (arrow) and no sclerotic rim **(B)**. Axial PET/CT fusion images showed moderate FDG uptake (arrow), with an SUVmax of 6.0 **(C)**. Sagittal T1WI shows mild compression of the T5 vertebral body, and the lesion exhibits hypointensity changes [**(D)**, arrow]. Contrast-enhanced T2WI shows significant enhancement of the lesion [**(E)**, arrow]. T2WI sagittal imaging shows a slight hyperintensity of the lesion [**(F)**, arrow]. Fat-suppression T2 sequence showing hyperintensity of the lesion [**(G)**, arrow].

**Figure 2:**
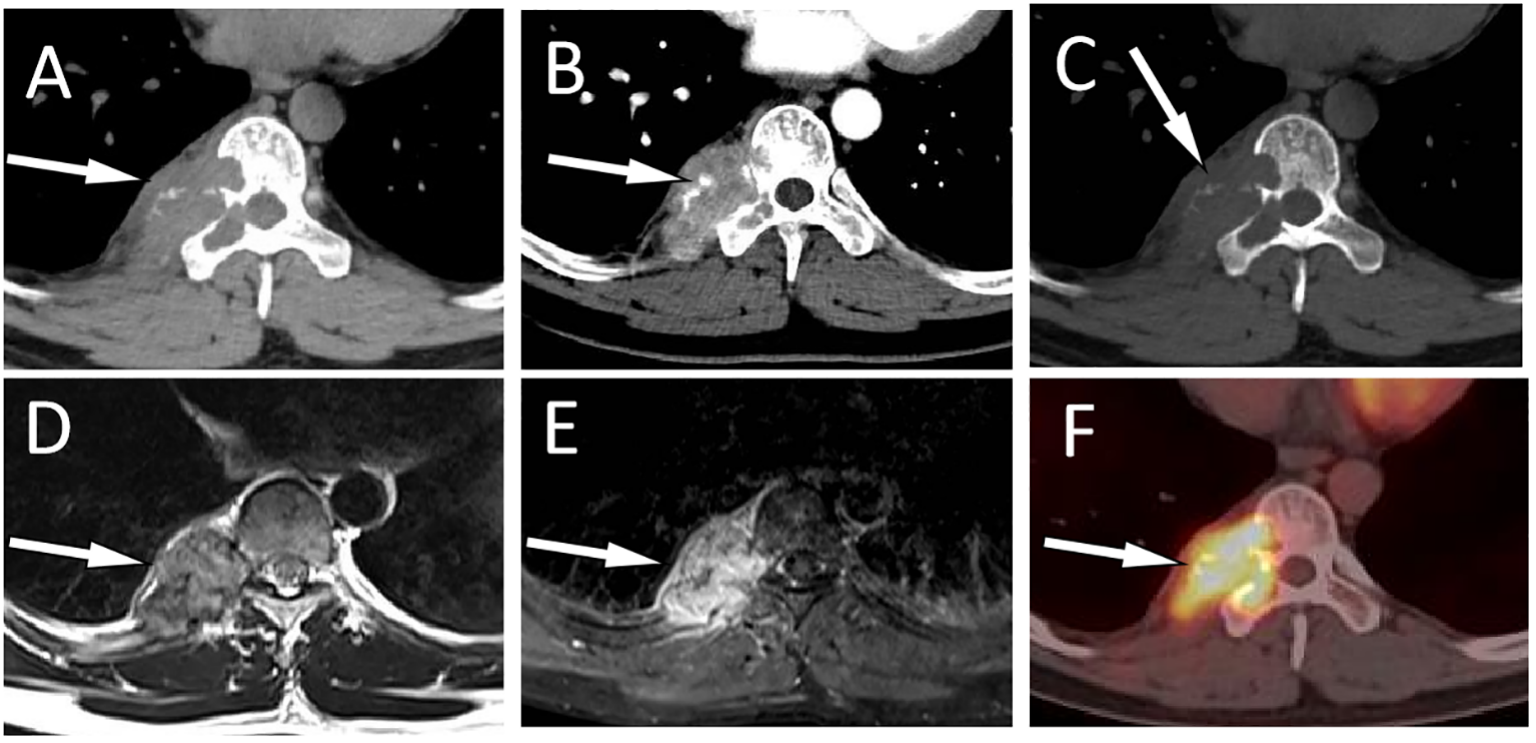
A 31-year-old man with EH (case 2): Axial CT soft tissue window displays a soft tissue mass extending toward the vertebral body and spinal canal centered around the right appendix of T9 [**(A)**, arrow]. Contrast-enhanced CT showed obvious enhancement of the mass, with blood supply vessel shadows visible inside [**(B)**, arrow]. The CT bone window shows expansive osteolytic bone destruction in the T9 vertebral body and the right accessory, with residual bone trabeculae visible inside (arrow) and no sclerotic rim **(C)**. T2WI sagittal imaging shows a slight hyperintensity of the lesion [**(D)**, arrow]. Fat-suppression T2 sequence showing hyperintensity of the lesion [**(E)**, arrow]. Axial PET/CT fusion images **(F)** showed moderate FDG uptake (arrow), with an SUVmax of 7.5.

The maximum diameter of the tumor ranges from 2.7 to 4.3. Among the five patients, four had lesions centered around the vertebral body and extending toward the accessories of the vertebral body (**Figure 1**), while only one had lesions centered around the accessories and extending toward the vertebral body (**Figure 2**). All lesions showed low-density expansive osteolytic bone destruction on CT, most lesions do not have a sclerotic rim (4/5), and three out of five lesions showed vertebral compression changes.

### MRI findings

On MRI, four lesions showed slightly hypointensity on T1WI, one lesion showed hypointensity, three lesions showed slight hyperintensity on T2WI, and two lesions showed isointensity. All lesions showed hyperintensity on fat suppression. In all five cases in which contrast-enhanced CT or/and MRI scanning was performed, an obvious enhancement pattern was noted.

### ^18^F-FDG PET/CT findings

All lesions exhibit higher ^18^F-FDG uptake than surrounding normal bone tissue, with a maximum standard uptake value (SUVmax) ranging from 5.4 to 9.6, mean standard uptake value (SUVmean) ranging from 3.0 to 4.0, and metabolic tumor volume (MTV) ranging from 13.8 to 44.5. The detailed imaging findings of all five cases are shown in **Table 2**.

**Table 2:** The imaging findings of thoracic spine epithelioid hemangioma patients.

Case	Location	MD	CT				MRI				CE	^18^F-FDG PET/CT			
			Density	SR	EO	VC	SH	T1WI	T2WI	FS		FDG uptake	SUVmax	SUVmean	MTV
1	T5 and accessories	3.0	Low	N	Y	Y	Heteroge-neous	hypointe-nsity	Slightly hyperin- tense	Hyperi-ntense	obv-ious	higher	6.0	3.1	22.0
2	T9 and accessories, right 9th rear rib	3.8	Low	N	Y	N	Homogen-eous	Slightly hypointe-nsity	Isointe-nse	Hyperi-ntense	obv-ious	higher	7.5	4.0	44.5
3	T2-3 and accessories	3.5	Low	Y	Y	Y	Heteroge-neous	Slightly hypointe-nsity	Slightly hyperin- tense	Hyperi-ntense	obv-ious	higher	9.6	3.4	21.2
4	T1 and accessories	4.3	Low	N	Y	Y	Heteroge-neous	hypointe-nsity	Isointe-nse	Hyperi-ntense	obv-ious	higher	7.9	3.0	28.5
5	T7 and accessories	2.7	Low	N	Y	N	Homogen-eous	Slightly hypointe-nsity	Slightly hyperin- tense	Hyperi-ntense	obv-ious	higher	5.4	3.6	13.8

CT, computed tomography; CE, contrast enhancement; EO, Expansile osteolysis; ^18^F-FDG, fluoro-18 fluorodeoxyglucose; FS, Fat suppression; SR, sclerotic rim; SH, Signal homogeneity; MD, maximum diameter; MTV, metabolic tumor volume; MRI, magnetic resonance imaging; N, no; PET, positron emission tomography; SUV, standardized uptake value; T1WI, T1-weighted imaging; T2WI, T2-weighted imaging; VC, Vertebral compression; Y, yes.

The preoperative images of cases 3–5 are shown in the **Supplementary Material** (**Supplementary Figures S1**-**S3**).

## Discussion

EH is an intermediate tumor that consists of epithelioid cells and endothelial cell differentiation. The etiology of this disease is currently not fully understood and may be related to trauma, infection, hormone levels, immune disorders, and environmental factors. A previous study revealed that elevated levels of vascular endothelial growth factor (VEGF) and interleukin-5 (IL-5) play an important role in the pathogenesis of EH (8). EH occurring in the bone is relatively rare, and research results show that spinal EH accounts for 6%–16% of skeletal EH, with thoracic spine EH being the most common (3, 6). The clinical manifestations of bone EH are non-specific, usually presented as local pain, swelling, and pathological fractures. Our study included five patients with thoracic spine EH, mainly middle aged and young, without gender preference. All patients sought medical help due to persistent chest and back pain for different periods of time, and two of them also had symptoms of limb numbness, which may be related to tumor compression or spinal cord invasion. Our patient’s serum tumor markers also showed no significant abnormalities.

The imaging findings, including CT, MRI, and ^18^F-FDG PET/CT of the thoracic spine EHs, have not been systematically reported previously. There are studies reporting that CT scans of thoracic spine EH typically present expansile osteolysis, with surrounding soft tissue masses accompanied by residual bone trabeculae, and rarely with a sclerotic rim (4, 9). Typical thoracic EH is prone to pathological compression fractures characterized by hypo- to isosignal on T1WI and hypersignal or slight hypersignal on T2WI. The contrast-enhanced CT or contrast-enhanced T1WI show moderate to significant enhancement, with uneven enhancement (10). In the current study, all lesions present as expansive low-density bone destruction shadows with soft tissue masses and no sclerotic rim. All lesions showed slightly low or low signal on T1WI, equal or slightly high signal on T2WI, and significantly high signal on fat-suppression sequence. These CT or MRI signs are basically consistent with literature reports. It is worth mentioning that two of our patients underwent contra-enhanced CT, which showed obvious blood-supplying arteries in the tumor, which exactly explained the reason for the obvious enhancement of the tumor. These blood-supplying arteries showed flow empty signals on both T1WI and T2WI. Four out of five patients underwent contrast-enhanced T1WI, all showing obvious enhancement. Considering some EH patients present with multiple lesions and more than one organ involved, PET/CT is a useful imaging method for evaluating it (11, 12). However, due to the rarity of this disease, ^18^F-FDG PET/CT features of the thoracic spine and even bones are rarely described in the literature. Our present study shows that PET/CT imaging of thoracic spine EH exhibits moderate ^18^F-FDG uptake, which may be related to its intermediate tumor nature.

The imaging differential diagnosis of EH includes giant cell tumors of the bone, hemangiomas, metastatic tumors, bone tuberculosis, and osteoblastoma. Giant cell tumor of the bone is also an intermediate tumor mostly occurring between the ages of 20 and 40 years. It is more common in the lower end of the femur and upper end of the tibia, but less common in the spine, which generally shows eccentric growth, and the dilatant osteolytic destruction is more obvious than EH (13). As the most common vasogenic tumor of the vertebral body, the typical CT findings of vertebral hemangiomas are reduced bone density in the affected vertebrae, with “fence-like” or “honeycomb” changes, mild swelling, and thinning of the bone cortex, lesions that may invade half or the entire vertebral body or annex, and occasional paravertebral or intravertebral soft tissue masses (14, 15). Compared with hemangiomas, EH presents more pronounced expansive osteolysis, more common soft tissue masses, and vertebral compression (9, 10). Metastatic bone tumors usually involve the posterior part of the vertebral body and pedicle, and the primary lesion can usually be found on PET/CT, which is not difficult to differentiate. Most spinal tuberculosis patients have a history of pulmonary tuberculosis, and the affected vertebrae are often characterized by irregular bone lysis destruction, narrowing or even disappearance of the vertebral space between the affected vertebrae, and paravertebral cold abscess and bone pontoon formation are also common. Contrast-enhanced CT or T1WI scans showed that the paravertebral abscess was a circular enhancement with relative specificity (16). Osteoblastoma is a rare benign osteoblastoma, most commonly seen in young people aged between 20 and 30 years and tends to occur in the spine and long bones. On CT, osteolytic bone destruction was also present, but there were hyperplastic reactions or sclerotic edges around the mass. Contrast-enhanced MRI showed that the interval of low signal within the mass had certain characteristics (17).

The diagnosis of EH depends on histopathological examination. Its main pathological features are microscopic proliferation of capillaries in lobulated arrangement, small capillaries can be seen around medium-sized mature blood vessels, and the endothelial cells of blood vessels are relatively enlarged, with abundant cytoplasm and eosinophilic appearance, protrusive into the vascular lumens like tombstones or boots, and very few vascular endothelial cells can be seen as vacuoles simulating the morphology of original blood vessels (2). In terms of immunohistochemistry, endothelial differentiation is an important criterion for diagnosing EHE. Tumor cells can express various vascular endothelial antigens, including CD31, CD34, ERG, and calponin (18). The tumor cells of the five patients included in our current study all positively expressed CD34, and four out of five positively expressed CD31 and ERG, which was consistent with previous literature reports.

The treatment of EH that occurs in the bone mainly involves local complete lesion resection, but some may experience local recurrence after surgery. Moreover, successful study has been reported in the treatment of EH with local injections of interferon-alpha, glucocorticoids, isotretinoin, or methotrexate (19). For the elderly and patients who cannot receive surgical treatment, radiofrequency ablation or laser therapy can be chosen, which has a low recurrence rate and minimal damage (20). All of our patients underwent complete resection of the mass, and one of them received local radiotherapy after surgery due to a slightly high Ki-67 index. No evidence of recurrence was found in all patients during the follow-up period.

The current work represents a preliminary analysis of thoracic spine EH imaging features, including CT, MRI, and ^18^F-FDG PET/CT, and presents some valuable findings. However, due to the rarity of the disease, the limitations of a limited number of thoracic spine EH cases should be taken into account when applying our research results.

In conclusion, thoracic spine EH usually manifests as chest and back pain, which can cause limb numbness when a mass compresses the spinal cord. The CT manifestation of thoracic EH is mild expansive osteolysis, usually without sclerotic rim. The lesion often involves both the vertebral body and accessories, accompanied by soft tissue masses, and residual bone trabeculae can be seen inside. On MRI, the mass shows slightly low signal on T1WI, slightly high signal or isosignal on T2WI, high signal on fat-suppression sequence, and significant enhancement on contrast-enhanced scanning. Moreover, the mass showed moderate ^18^F-FDG uptake on PET/CT.

## Data availability statement

The original contributions presented in the study are included in the article/**Supplementary Material**. Further inquiries can be directed to the corresponding authors.

## Ethics statement

The studies involving humans were approved by the institutional review board of the Affiliated Hospital of Zunyi Medical University. The studies were conducted in accordance with the local legislation and institutional requirements. Written informed consent for participation was not required from the participants or the participants’ legal guardians/next of kin in accordance with the national legislation and institutional requirements. Written informed consent was obtained from the individual(s) for the publication of any potentially identifiable images or data included in this article.

## Author contributions

XH: Conceptualization, Data curation, Formal analysis, Resources, Writing – original draft. XL: Investigation, Methodology, Validation, Writing – original draft. ZX: Data curation, Software, Validation, Writing – original draft. DL: Investigation, Methodology, Project administration, Writing – original draft. JC: Data curation, Investigation, Resources, Writing – review & editing. PW: Investigation, Project administration, Supervision, Visualization, Writing – review & editing.
