# Supplementary material for: CT, MRI, and PET/CT imaging features of thoracic spine epithelioid hemangioma: a retrospective observational study
Source: Front Oncol. 2024 Jun 19;14:1296401. doi: 10.3389/fonc.2024.1296401 (PMC11220569; doi:10.3389/fonc.2024.1296401)

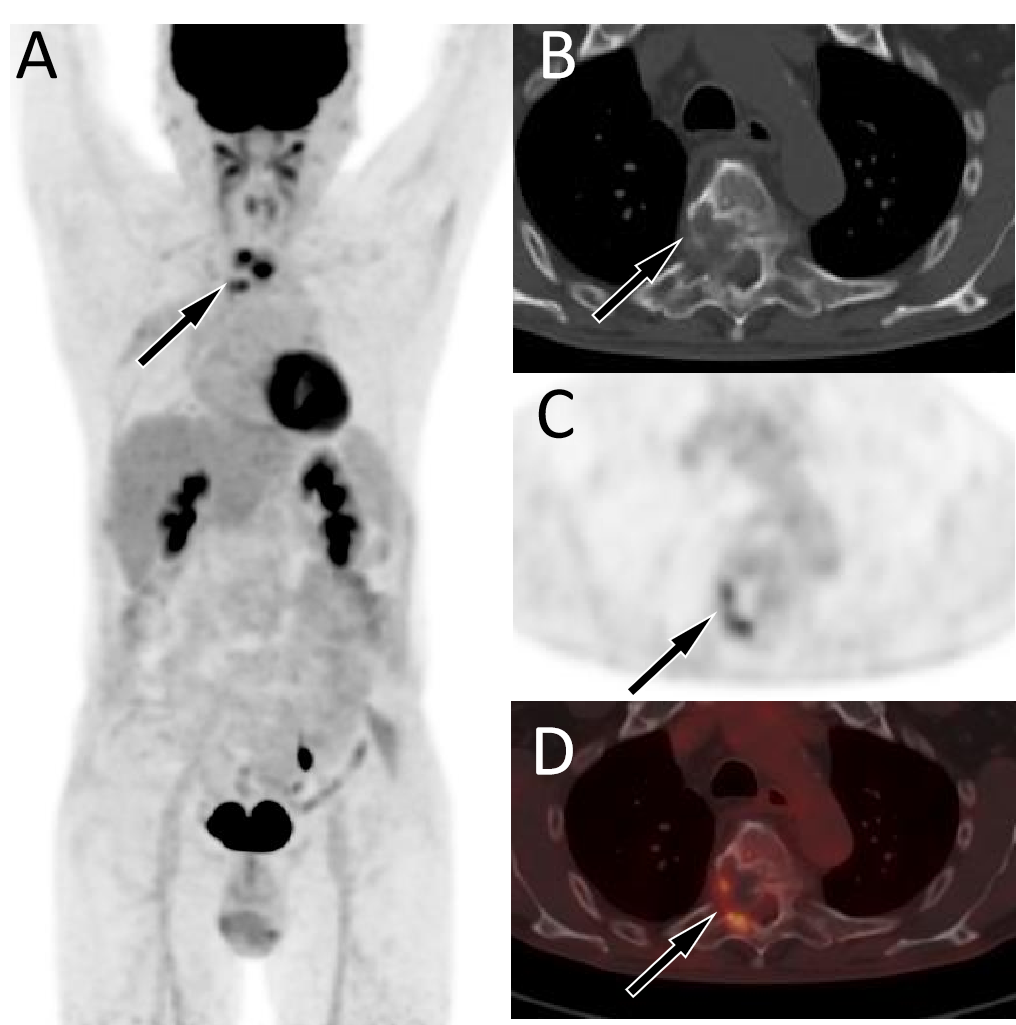


**Fig. S1**. A 56-year-old man with EH (case 3): The maximum intensity projection shows an increased ^18^F-FDG lession in the upper thoracic vertebra (**A**, arrow); The CT bone window shows expansive osteolytic bone destruction in the T1 vertebral body and the right accessory, with residual bone trabeculae visible inside and sclerotic rim (**B**, arrow). Axial PET (**C**) and PET/CT fusion images (**D**) showed increased uptake of ^18^F-FDG in this lesion, with a SUVmax of 9.6.


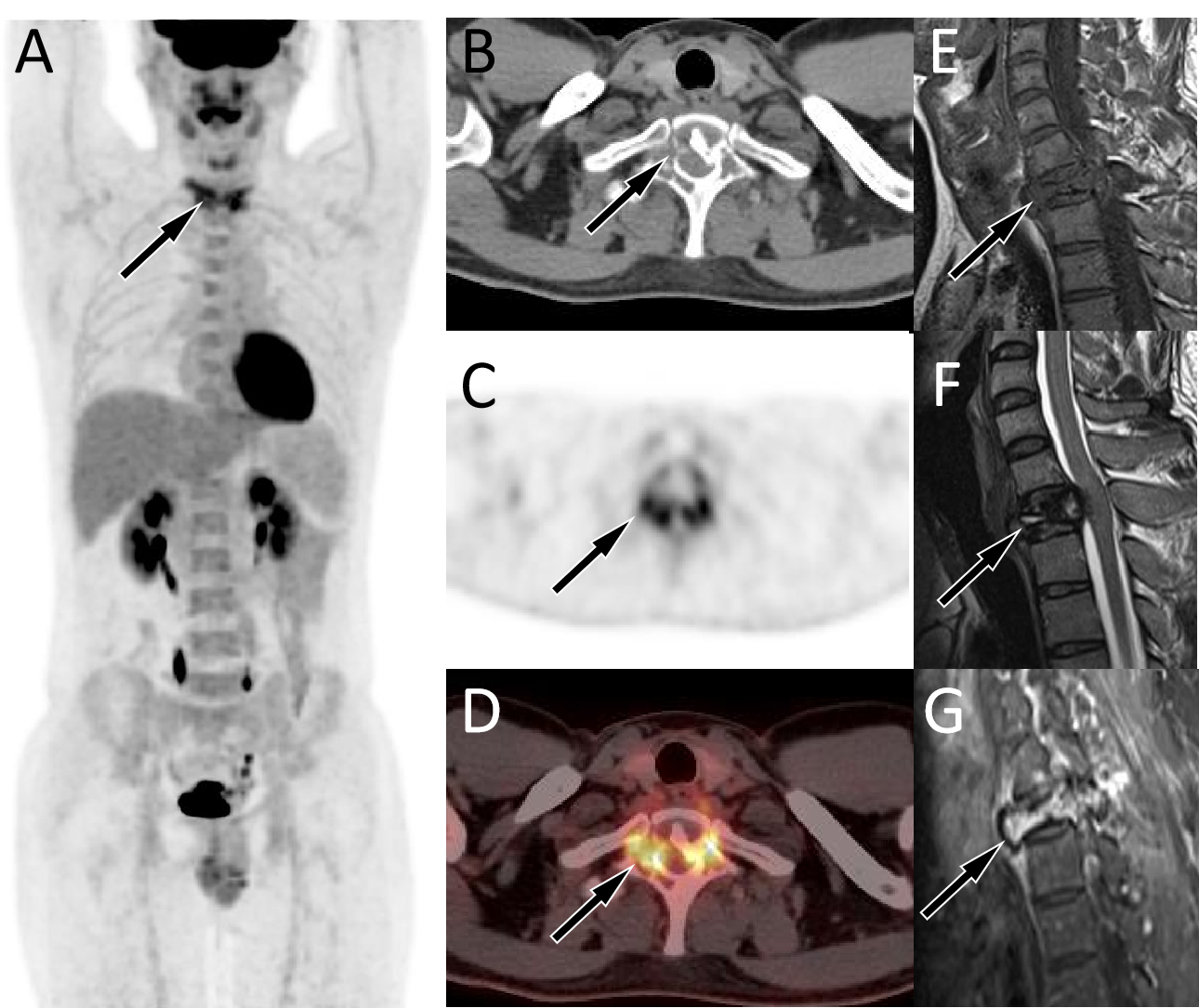


**Fig. S2**. A 31-year-old man with EH (case 4): The maximum intensity projection shows an increased ^18^F-FDG lession in the upper thoracic vertebra (**A**, arrow); The axial CT shows a soft tissue mass extending towards the periphery of the vertebral body and bilateral accessories as well as spinal canal with T1 as the center (**B**, arrow). Axial PET (**C**) and PET/CT fusion images (**D**) showed increased uptake of ^18^F-FDG in this lesion, with a SUVmax of 7.9. Sagittal T1WI shows the lesion presents [hypoi](javascript:;)ntensity (**E**, arrow). T2WI sagittal imaging shows a sightly [hyperintensity](javascript:;) of the lesion, the signal is uneven (**F**, arrow). Contrast enhanced T1WI shows significant enhancement of the lesion (**G**, arrow).


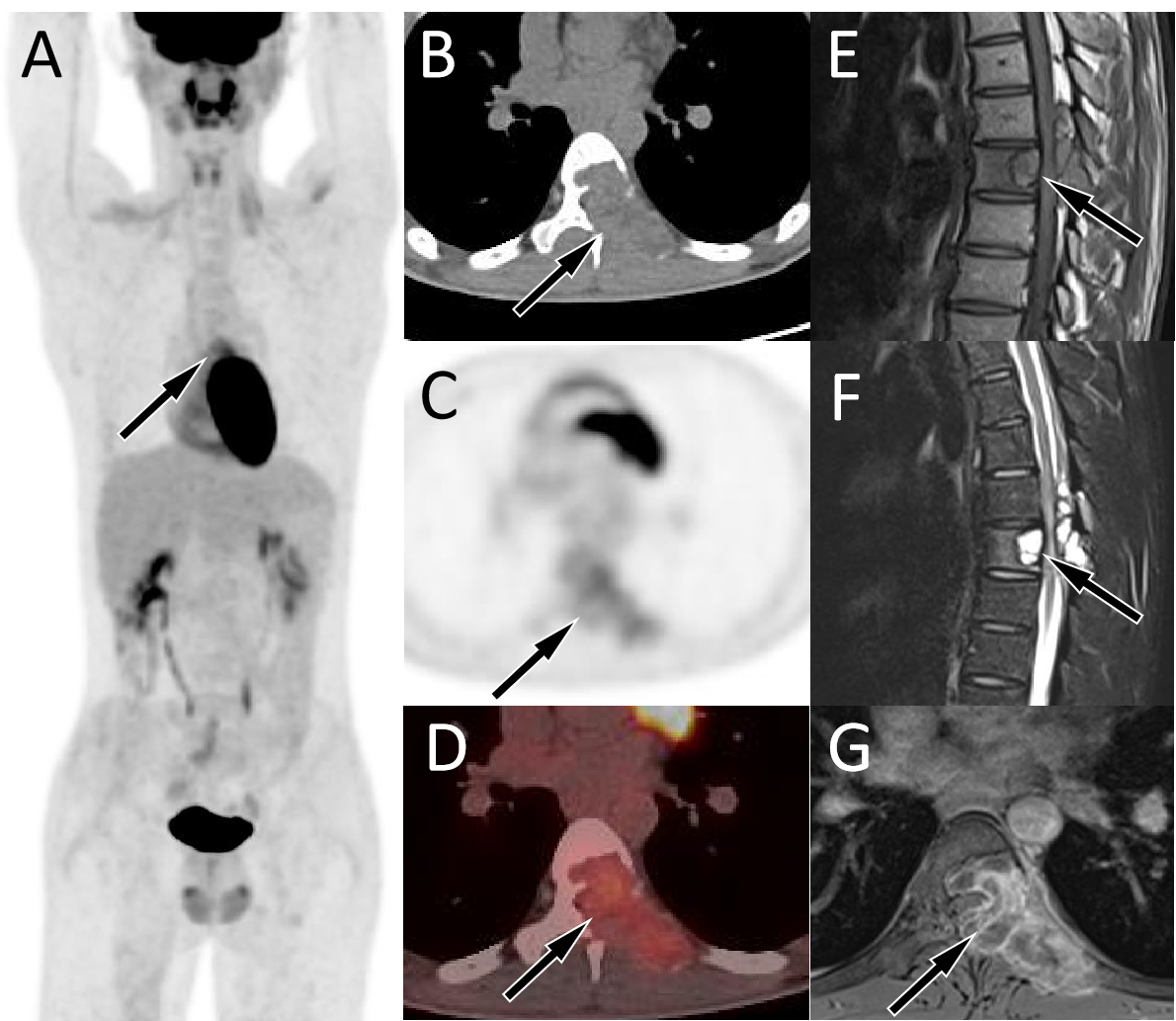


**Fig. S3**. A 23-year-old man with EH (case 5): The maximum intensity projection shows an increased ^18^F-FDG lession in the middle thoracic vertebra (**A**, arrow); The axial CT shows a soft tissue mass extending towards the periphery of the vertebral body and the left accessory as well as spinal canal with T7 as the center (**B**, arrow). Axial PET (**C**) and PET/CT fusion images (**D**) showed increased uptake of ^18^F-FDG in this lesion, with a SUVmax of 5.4. Sagittal T1WI shows the lesion presents [isoi](javascript:;)ntensity (**E**, arrow). T2WI sagittal imaging shows a [hyperintensity](javascript:;) of the lesion (**F**, arrow). Contrast enhanced T1WI shows significant enhancement of the lesion (**G**, arrow).


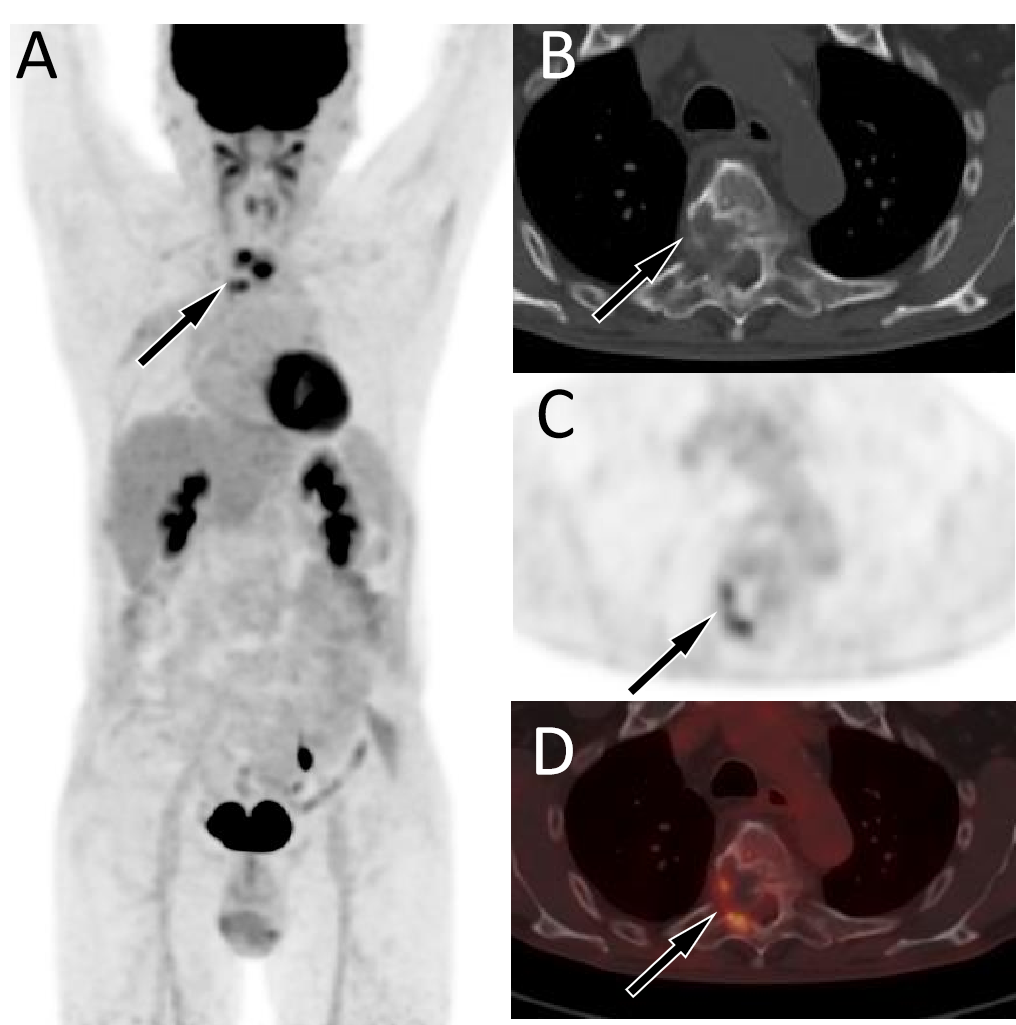

Supplement: Supplementary file 1 [file DataSheet_1.doc]
